# Biology and Comparative Mitogenomics of *Psyttalia carinata*, Newly Recorded From China as a Parasitoid of *Rhagoletis batava*

**DOI:** 10.1002/ece3.74415

**Published:** 2026-10-15

**Authors:** Kailiang Li, Zhiqiang Ge, Zhenyu Zhang, Wenchao Guo, Hongying Hu

**Affiliations:** ^1^ College of Life Science and Technology, Xinjiang Key Laboratory of Biological Resources and Genetic Engineering Xinjiang University Urumqi China; ^2^ Xinjiang Key Laboratory of Agricultural Biosafety Institute of Plant Protection, Xinjiang Uygur Autonomous Region Academy of Agricultural Sciences Urumqi Xinjiang China; ^3^ Key Laboratory of Integrated Pest Management on Crops in Northwestern Oasis, Ministry of Agriculture and Rural Affairs Urumqi Xinjiang China

**Keywords:** gene rearrangement, mitochondrial genome, new record for China, parasitoid wasp, *Rhagoletis batava*

## Abstract

Reliable species identification, host confirmation, and biological information are essential for evaluating parasitoids in natural‐enemy systems. We integrated regional surveys, taxonomic evidence, direct host verification, life‐history observations, and comparative mitogenomics to characterize *Psyttalia carinata*, newly recorded from China as a parasitoid of the sea buckthorn fruit fly, *Rhagoletis batava* . Surveys covered 30 sites across Xinjiang. Species identity was established by morphological diagnosis and expert verification, while host association was confirmed by parasitoid emergence and dissection of puparia formed by larvae from sea buckthorn fruits; all emerged tephritids were *R. batava* . Of 8260 puparia from Altay and Akqi, 595 yielded adults or contained immature *P. carinata* , giving an overall apparent parasitism of 7.2%. *Psyttalia carinata* developed as a solitary larval–pupal koinobiont endoparasitoid and overwintered as a pupa within the host puparium. Malaise trap monitoring across two field seasons detected one concentrated annual period of adult activity in the Altay population, consistent with a univoltine life cycle. A factorial experiment with 720 adults showed that longevity decreased with increasing temperature, was markedly extended by honey feeding, and was generally greater in females than males. Illumina sequencing yielded a complete 15,633‐bp mitochondrial genome containing 37 genes and 82.7% A + T. Across five available *Psyttalia* mitogenome assemblies, variation was concentrated in tRNA gene order. *P. carinata* and *P. incisi* shared the same complete mitochondrial gene order, including nad4L–trnT–trnP–nad6, whereas *P. concolor* , *P. humilis* , and *P. lounsburyi* shared nad4L–trnP–trnT–nad6. However, phylogenetic analyses placed *P. carinata* as sister to the latter three‐species clade rather than to *P. incisi*, indicating that identical gene order did not correspond to the closest sequence‐based relationship. These findings provide an integrated biological and mitogenomic reference for *P. carinata* and a basis for evaluating its ecological role in the *R. batava* system.

## Graphical abstract

*Psyttalia carinata* is newly recorded from China and was directly confirmed as a solitary larval–pupal koinobiont endoparasitoid of the sea buckthorn fruit fly, *Rhagoletis batava* . Field surveys, host rearing, dissections, and longevity experiments characterized its occurrence and biology, while Illumina sequencing yielded a complete 15,633‐bp mitochondrial genome. Comparative gene‐order and phylogenetic analyses revealed that variation among *Psyttalia* species was concentrated mainly in tRNA genes and that local gene‐order similarity did not necessarily reflect the closest sequence‐based relationship.

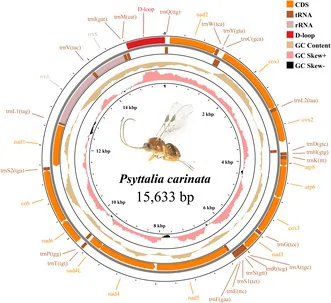

## 1 Introduction

Larvae of fruit‐infesting tephritid flies develop within fruits, reducing fruit yield and quality while remaining difficult to reach with conventional control measures. Parasitoid wasps can suppress these concealed herbivores and are therefore important components of natural and managed ecosystems (Abram et al. 2019; Hawkins 1994). Braconidae is one of the most species‐rich parasitoid families within Hymenoptera, with more than 20,000 described species worldwide (Quicke 2014; Yu et al. 2016). Many members of Opiinae are koinobiont endoparasitoids of dipteran larvae, and species of *Psyttalia* Walker are particularly associated with fruit‐infesting tephritids (Carmichael et al. 2005; Li et al. 2013; van Achterberg 2023; Wharton 1997). Because their hosts continue to develop after parasitism, successful parasitoid development depends on host age, resource availability, and phenological synchrony (Pennacchio and Strand 2006). Reliable identification, distribution records, and life history information are therefore essential for evaluating their ecological roles and biological control potential (Heimpel et al. 2021; Seehausen et al. 2020).

Sea buckthorn, *Hippophae rhamnoides* L., is a woody plant of substantial ecological, nutritional, and economic importance across temperate Eurasia. Its actinorhizal root nodules establish a nitrogen‐fixing symbiosis with *Frankia*, enabling its use in vegetation restoration, soil improvement, and the rehabilitation of marginal lands. Its fruits are rich in nutrients and are processed into foods, oils, and products of high economic value (Ma et al. 2023; Wu et al. 2022; Yu et al. 2024). The sea buckthorn fruit fly, *Rhagoletis batava* (Hering, 1958), is a highly specialized tephritid for which sea buckthorn is currently the only known larval host. Females oviposit into developing fruits, and the resulting larvae feed within the pulp, causing softening, deformation, discoloration, and desiccation and thereby substantially reducing fruit yield, market quality, and processing value (Aleknavičius and Būda 2019; Tartanus et al. 2025). The severity of damage varies among years, regions, and sea buckthorn cultivars. During outbreak years, or in susceptible cultivars and orchards lacking effective management, fruit infestation may exceed 80%, and nearly all marketable fruit may be lost under severe conditions (Blažytė‐Čereškienė et al. 2022; Zeynalov 2018). Nevertheless, the composition of native parasitoid assemblages associated with this pest, their patterns of host use, and their seasonal occurrence remain poorly documented. Preliminary observations beginning in 2022 documented recurrent damage by *R. batava* in Xinjiang sea buckthorn habitats and prompted investigation of its native parasitoids as potential biological control resources.

*Psyttalia carinata* (Thomson, 1895) has been recorded from several parts of the Palaearctic region and is known to parasitize tephritid flies belonging to the genera *Rhagoletis*, *Myoleja*, *Chetostoma*, and *Carpomya*. Morphologically, it resembles the congener *P. concolor* , but the two species can be distinguished using a combination of characters involving the shape of the head behind the compound eyes, body coloration, and forewing venation (Wu et al. 2016). Regional taxonomic revisions, faunal catalogs, and major public databases contain no reliable formal record of *P. carinata* from China. Its verified host use, developmental biology, overwintering, local parasitism, and seasonal occurrence in China also remain undocumented. Confirming its occurrence and establishing a direct association with *R. batava* therefore have both biogeographic and ecological significance.

In addition to nucleotide sequence information, mitochondrial genomes can preserve evolutionary signals through their gene content, transcriptional orientation, and gene order. A typical insect mitochondrial genome contains 13 protein‐coding genes, 22 tRNA genes, two rRNA genes, and one major non‐coding control region, although the stability of gene arrangement varies substantially among lineages (Cameron 2014, 2025). Hymenoptera is among the insect orders with the highest frequency of mitochondrial gene rearrangement. Particularly within Apocrita, translocations, inversions, inverted transpositions, and localized rearrangements of gene clusters can arise at relatively shallow taxonomic levels. Regions adjacent to *cox2–atp8* and *nad3–nad5* have been identified as rearrangement hotspots, with multiple derived arrangements occurring within the same local genomic regions (Dowton and Austin 1999; Dowton et al. 2003). These changes are concentrated primarily in tRNA genes, possibly because of their short length and the comparatively weak functional constraints imposed on their genomic positions. A comparative analysis of 67 tRNA rearrangements in Hymenoptera showed that some shared changes may serve as lineage‐specific characters, whereas identical arrangements may also arise independently in different lineages (Dowton et al. 2009). Tandem duplication followed by the random loss of redundant copies, replication errors, and intramolecular recombination have all been proposed as mechanisms underlying mitochondrial gene rearrangement, although local inversions are often difficult to explain using a simple duplication–deletion model alone (Cameron 2025; Dowton et al. 2003; Wei et al. 2010). Gene order can provide structural characters of phylogenetic value, but parallel rearrangements, reversals, and retention of ancestral arrangements may generate incongruence with phylogenies inferred from sequence data (Cameron 2014, 2025; Dowton et al. 2009). Although parasitoid life histories have been discussed as possible contributors to mitochondrial genome evolution, broader sampling has not identified a single general pattern across Hymenoptera (Castro et al. 2002; Dowton et al. 2009). Comparative analyses should therefore first identify conserved and variable arrangements and evaluate their agreement with sequence phylogenies; ecological explanations require broader taxonomic sampling and explicit comparative tests.

Comparative mitogenomic studies of Braconidae have similarly shown that the arrangement of protein‐coding and rRNA genes is generally conserved, whereas tRNA genes account for most rearrangements. Both nucleotide sequences and gene order can provide phylogenetic signal at some taxonomic levels, although strong compositional bias, rapid sequence evolution, and recurrent rearrangement may reduce the stability of phylogenetic inference (Jasso‐Martínez et al. 2022; Mao et al. 2015; Tang et al. 2019; Wei et al. 2010). Within *Psyttalia*, Powell et al. (2020) reported the complete mitochondrial genome of *P. concolor* and near‐complete mitogenomes of *P. humilis* and *P. lounsburyi*, revealing several rearrangements involving tRNA genes. The complete mitochondrial genome of *P. incisi* was subsequently reported by Yang et al. (2022). However, mitogenomic resources for Psyttalia remain limited, and neither the mitochondrial gene arrangement of *P. carinata* nor its relationship to sequence‐based phylogeny within the genus has been evaluated. Obtaining the complete mitochondrial genome of *P. carinata* therefore not only expands molecular sampling within *Psyttalia* but also provides a new comparative basis for identifying shared rearrangements and localized gene‐order divergence and for testing the congruence between gene‐order signals and mitochondrial sequence phylogenies.

In this study, we surveyed the principal sea buckthorn distribution and cultivation areas of Xinjiang and monitored a fixed Altay site across two field seasons to determine whether the recovered parasitoid was *P. carinata* and represented a new record for China. Morphological diagnosis, expert verification, host emergence, dissection, and laboratory rearing were used to confirm species identity and host association and to characterize development within the host, apparent parasitism, overwintering, seasonal occurrence, and adult longevity. Because *R. batava* is univoltine in the study region, we predicted a corresponding annual cycle in *P. carinata* . We also predicted that higher temperature would shorten adult longevity, whereas access to sugar would prolong survival. At the molecular level, we assembled and annotated the complete mitochondrial genome of *P. carinata* , compared its gene arrangement with the four other available *Psyttalia* assemblies, and inferred its phylogenetic placement from the 13 mitochondrial protein‐coding genes. We predicted that differences in gene arrangement would be concentrated in tRNA genes and tested whether gene arrangement agreed with the phylogeny inferred from sequence data. Together, these analyses establish an integrated taxonomic, biological, and mitogenomic reference for *P. carinata* in China, providing a foundation for evaluating its ecological role and potential contribution to the biological control of *R. batava* , as well as for future comparative studies of mitochondrial genome evolution within *Psyttalia*.

## 2 Materials and Methods

### 2.1 Study Area and Field Sampling

Surveys of the sea buckthorn fruit fly, *Rhagoletis batava* (Hering, 1958), and its parasitoids were conducted from July 2023 to September 2025 across the principal natural distribution and cultivation areas of sea buckthorn in Xinjiang, China. The regional survey comprised 30 sampling sites: nine in Altay Prefecture, eight in Aksu Prefecture, four in Kizilsu Kirgiz Autonomous Prefecture, six in Kashgar Prefecture, and one each in Urumqi, Ili Kazakh Autonomous Prefecture, and Changji Hui Autonomous Prefecture. The surveyed habitats included natural sea buckthorn stands, ecological plantations, and cultivated sea buckthorn orchards. At each site, three plots were established, and 30 sea buckthorn plants were examined in each plot using a five‐point sampling design, yielding a total of 2700 plants. For each site, the prefecture‐ and county‐level administrative divisions, survey locality, geographic coordinates, elevation, sea buckthorn stand type, and detection status of *R. batava* and *P. carinata* are provided in Table S1. Fruits were inspected for oviposition punctures, abnormal discoloration, softening, collapse, and signs of larval feeding. Fruits or fruit‐bearing shoots showing typical damage symptoms were labeled by locality and sampling date, placed in ventilated mesh bags, and transported to the laboratory for rearing.

A continuously monitored study site was established in Woyimoke Town, Burqin County, Altay Prefecture (47.71956° N, 86.920122° E; 440.74 m a.s.l.). The site covered approximately 12 ha and consisted of an artificially established ecological plantation of *Hippophae rhamnoides* subsp. *mongolica* “Shenqiuhong.” The trees were approximately 10–12 years old, and no chemical insecticides were applied during the study period. Surveys were conducted at intervals of approximately 20 days from May 2024 to September 2025. On each sampling date, six rows were selected, and 20 trees per row were systematically examined, giving a total of 120 trees. Survey rows and individual trees were reselected on each sampling date rather than permanently marked. The canopy of each tree was divided into four cardinal directions—east, south, west, and north—and two vertical strata—upper and lower—resulting in eight canopy sectors. From each sector, one 10–20‐cm fruit‐bearing shoot containing a relatively high number of damaged fruits was collected and transported to the laboratory.

To monitor the seasonal occurrence of host and parasitoid adults, two Malaise traps were permanently installed at the fixed study site from April to November in both 2024 and 2025. Collection bottles were replaced every 10 days, and adults of *R. batava* and *P. carinata* were identified and counted separately. Trap positions, collection intervals, and identification criteria were kept consistent between years to compare the occurrence periods, concentrated activity periods, and relative timing of abundance peaks of the two species. Soil sampling was conducted concurrently during May–June and August of each year to survey overwintering puparia formed in the preceding year and newly formed puparia from the current year, respectively. At each sampling event, 100 sea buckthorn trees were selected using a five‐point sampling design. Based on previous evidence that *R. batava* puparia are concentrated near the trunk and in the upper soil layers (Zhang et al. 2017), soil was collected within a 20‐cm radius of the trunk to a depth of 0–40 cm to improve puparial recovery and obtain sufficient material for rearing and dissection. The sample from each tree was processed independently. Soil samples were sequentially sieved through meshes of 2.5 × 2.5 mm and 1 × 1 mm, and tephritid puparia were recovered manually. Samples were labeled by tree and sampling date and remained separate during soil processing and puparial recovery. Recovered puparia were subsequently pooled within the same locality‐by‐date collection batch for laboratory rearing, emergence observation, and dissection.

Both the regional survey and fixed‐site monitoring were designed specifically to investigate *R. batava* and its parasitoids in sea buckthorn habitats. The fixed Altay site was selected for detailed investigation of host use, seasonal occurrence, and overwintering by *P. carinata* because it combined recurrent host occurrence with a relatively large study area, absence of chemical insecticide applications, accessibility, and suitability for repeated sampling. Accordingly, the present study characterizes *P. carinata* in association with *R. batava* in sea buckthorn habitats in Xinjiang and does not evaluate host associations outside this study system.

### 2.2 Laboratory Rearing, Host‐Association Confirmation, and Biological Observations

After mature larvae exited the fruits, they were collected manually and allowed to pupariate. The resulting puparia were grouped by locality and collection batch. Puparia belonging to the same locality‐by‐batch group were pooled in glass Petri dishes, with approximately 500 individuals per dish; batches containing fewer than 500 puparia were maintained as separate dishes. Each dish therefore contained puparia from only one locality and one collection batch. The puparia were not reared individually, and emergence records were maintained at the dish and collection‐batch levels. No soil, sand, or other substrate was added. Each dish was sealed with plastic film perforated with ventilation holes and maintained at 15°C, 60% relative humidity, and a photoperiod of 14 h light:10 h dark. Emergence of host flies and parasitoids was checked daily, and observations continued until August of the following year.

A total of 8260 *R. batava* puparia were maintained under long‐term rearing conditions, including 5400 from Altay and 2860 from Akqi. At the end of the observation period, each remaining unemerged puparium was opened and examined separately, and the presence and developmental stage of any immature tephritid or parasitoid were recorded. Because puparia were pooled within locality‐by‐batch groups, emerged parasitoids could be assigned to their corresponding locality and collection batch.

Independent subsamples of host larvae and puparia collected at different sampling times were dissected at intervals of approximately 7 days, with approximately 30 individuals examined on each occasion. In total, more than 900 host larvae and puparia were dissected. For each individual, the presence, position, and developmental stage of the parasitoid within the host were recorded. The dissections were used to characterize the mode of parasitism, development across the host larval and puparial stages, and overwintering stage of *P. carinata* . The dissection series was discontinued once these target biological characteristics had been consistently established through repeated observations. These specimens were independent of and were not included among the 8260 puparia used for long‐term rearing and apparent‐parasitism calculations. Apparent parasitism in the collected host samples was calculated as follows: apparent parasitism (%) = (number of emerged *P. carinata* adults + number of unemerged puparia containing parasitoid immatures)/total number of host puparia examined × 100.

### 2.3 Species Identification, Morphometric Measurements, and Voucher Deposition

Adult parasitoids obtained in this study were identified morphologically primarily using the key to *Psyttalia* and the species‐level diagnostic characters provided by Wu et al. (2016). Relevant regional taxonomic revisions, faunal checklists, taxonomic catalogs, and publicly accessible biodiversity databases were further consulted to cross‐check taxonomic information and previous distribution records. Representative specimens were subsequently examined and independently verified by Dr. Cornelis van Achterberg.

Because sufficient specimen series were available from both Altay in northern Xinjiang and Akqi in southern Xinjiang, these geographically separated populations were selected for morphometric comparison. As the principal species‐level diagnostic characters of *P. carinata* are based on females, five representative females from each locality were measured using a Nikon SMZ25 stereomicroscope. Measurements included body length and key diagnostic and proportional characters of the head, antennae, forewing venation, first metasomal tergite, hind leg, and ovipositor sheath. Complete information on the measured characters, character definitions, sample sizes, and measurement ranges is provided in Table S2. Pinned voucher specimens of both sexes were deposited in the Insect Collection of Xinjiang University (ICXU), Urumqi, China.

### 2.4 Effects of Temperature, Diet, and Sex on Adult Longevity

A fully factorial experiment was conducted to evaluate the effects of temperature, diet, and sex on the adult longevity of *P. carinata* . The experiment comprised two sex levels (female and male), four temperature levels (15°C, 20°C, 25°C, and 30°C), and three dietary treatments (water, 15% honey solution, and 30% honey solution), resulting in 24 treatment combinations. Thirty adults were assigned to each treatment combination, with each individually maintained adult constituting an independent experimental unit, giving a total sample size of 720 individuals. Experimental adults originated from multiple emergence dates and rearing batches from Altay and Akqi; geographic origin and emergence batch were not included as experimental factors.

Newly emerged adults less than 12 h old were selected, sexed immediately, and individually confined in glass tubes (15 mm in diameter × 90 mm in length), with the tube openings sealed using cotton plugs. The 15% and 30% honey solutions were prepared on a volume/volume basis. Water or honey solution was provided ad libitum on cotton balls moistened with the respective treatment and replaced daily. The four temperature treatments were maintained under controlled‐environment conditions with a photoperiod of 14 h light:10 h dark and a relative humidity of 60% ± 5%. Actual temperature and relative humidity were independently monitored throughout the experiment to verify that the target environmental conditions were maintained. Thereafter, survival was assessed daily at 10:00, 16:00, and 22:00. An individual was considered dead when it showed no response to external stimulation, and the time at which death was first detected was recorded as the time of death. Longevity was initially recorded in hours and subsequently converted to days by dividing by 24.

### 2.5 DNA Extraction, Sequencing, Mitochondrial Genome Assembly, and Annotation

One adult female *P. carinata* collected from Altay and confirmed by morphological identification was selected for genomic DNA extraction. Total genomic DNA was extracted using the Ezup Column Animal Genomic DNA Purification Kit (Sangon Biotech [Shanghai] Co. Ltd., China) according to the manufacturer's instructions. DNA integrity was assessed by electrophoresis on a 0.75% agarose gel containing 0.5 μg/mL ethidium bromide. The DNA sample that passed quality assessment was submitted to Sangon Biotech (Shanghai) Co. Ltd. for library preparation and sequencing.

A paired‐end whole‐genome shotgun library with an average insert size of approximately 350 bp was constructed and sequenced on an Illumina NovaSeq X Plus platform in PE150 mode, generating approximately 6 Gb of raw data in FASTQ format. Raw reads were quality‐filtered using fastp v0.23.4 (Chen et al. 2018) to remove adapter‐contaminated reads, low‐quality reads, reads containing a high proportion of ambiguous nucleotides, and highly duplicated reads. The resulting high‐quality reads were used for downstream assembly.

The mitochondrial genome was assembled de novo and provisionally annotated using MitoZ v3.6 (Meng et al. 2019). The filtered reads were mapped back to the preliminary assembly, and continuity of sequence coverage and potential ambiguous sites were examined in Geneious Prime v2025.1.2. Annotations of protein‐coding genes, rRNA genes, and tRNA genes were independently checked using MITOS2 (Bernt et al. 2013; Donath et al. 2019), and tRNA genes and their secondary structures were predicted using tRNAscan‐SE v2.0 (Chan et al. 2021). Gene boundaries were manually refined in Geneious Prime based on open reading frames, the positions of adjacent genes, and homologous sequences from closely related species. The final mitochondrial genome sequence was deposited in GenBank under accession number PX579120. A circular map of the mitochondrial genome was generated using the Proksee platform (Grant et al. 2023).

### 2.6 Mitochondrial Genome Characteristics and Gene‐Order Comparisons

Gene content, transcriptional orientation, intergenic spacers and overlaps, nucleotide composition, start and stop codons of the 13 protein‐coding genes, codon usage, and relative synonymous codon usage were analyzed using Geneious Prime v2025.1.2, MEGA v12 (Kumar et al. 2024), and PhyloSuite v2 (Zhao et al. 2025). Nucleotide compositional skews were calculated following Perna and Kocher (1995) as AT‐skew = (*A* − *T*)/(*A* + *T*) and GC‐skew = (*G* − *C*)/(*G* + *C*). Gene‐order comparisons included the five currently available *Psyttalia* mitogenome assemblies: *P. carinata* (PX579120), *P. incisi* (OK413636), *P. concolor* (MW279212), *P. humilis* (MW279213), and *P. lounsburyi* (MW279214). The mitogenomes of *P. carinata* , *P. incisi*, and *P. concolor* are complete, whereas those of *P. humilis* and *P. lounsburyi* are near‐complete because the A + T‐rich region and adjacent trnQ region were not fully recovered (Powell et al. 2020). Gene‐order comparisons were therefore based on homologous regions represented in the respective assemblies. The gene arrangements of *Daphnia pulex* (NC_000844) and *Formica fusca* (LN607805) were included as comparative references. The assemblies were standardized relative to cox1 and oriented consistently for comparison. The relative positions and transcriptional directions of the recovered genes were compared, with particular attention to the regions surrounding cox2–atp8, nad2–cox1, and nad4L–nad6. A change in gene position without a change in transcriptional orientation was classified as a translocation, a change in orientation without positional displacement as an inversion, and a change in both position and orientation as an inverted transposition. The mitochondrial gene arrangement of *D. pulex* was used as a widely adopted pancrustacean reference, whereas that of *F. fusca* served as a relatively conserved, putatively plesiomorphic reference within Apocrita. Neither species was regarded as representing the actual ancestor of Hymenoptera.

### 2.7 Phylogenetic Analyses

A PCG123 dataset comprising all three codon positions of the 13 mitochondrial protein‐coding genes was constructed. The analysis included 23 species of Braconidae, with *Diadegma semiclausum* (NC_012708) and *Gotra octocincta* (NC_072299) from Ichneumonidae selected as outgroups, resulting in a total of 25 taxa (Table 1). GenBank files for all included species were downloaded from the NCBI database and imported into PhyloSuite v2 (Zhao et al. 2025), from which the 13 protein‐coding genes were extracted separately. Each gene was initially aligned using MAFFT v7.526 (Katoh and Standley 2013), after which the codon reading frames were refined using MACSE v2 (Ranwez et al. 2011). Poorly aligned regions were removed using trimAl v2 (Capella‐Gutiérrez et al. 2009), and the resulting alignments were concatenated into a combined dataset using FASconCAT‐G v1.04 (Kück and Longo 2014).

**TABLE 1:** Taxa and GenBank accession numbers included in the phylogenetic analyses.

Family	Subfamily	Species	GenBank accession number
Ichneumonidae	Campopleginae	*Diadegma semiclausum*	NC_012708
Ichneumonidae	Cryptinae	*Gotra octocincta*	NC_072299
Braconidae	Alysiinae	*Asobara japonica*	NC_045903
Braconidae	Aphidiinae	*Binodoxys acalephae*	NC_073545
Braconidae	Aphidiinae	*Binodoxys communis*	NC_069846
Braconidae	Aphidiinae	*Aphidius colemani*	NC_069199
Braconidae	Braconinae	*Euurobracon yokahamae*	NC_064332
Braconidae	Braconinae	*Habrobracon hebetor*	MN842279
Braconidae	Braconinae	*Cyanopterus ninghais*	OR525636
Braconidae	Braconinae	*Virgulibracon endoxylaphagus*	NC_062620
Braconidae	Cheloninae	*Chelonus formosanus*	NC_060869
Braconidae	Cheloninae	*Chelonus munakatae*	OQ885490
Braconidae	Doryctinae	*Spathius agrili*	NC_014278
Braconidae	Meteorinae	*Meteorus pulchricornis*	NC_053259
Braconidae	Microgastrinae	*Apanteles gelechiidivoris*	NC_082146
Braconidae	Microgastrinae	*Microplitis manilae*	NC_079819
Braconidae	Microgastrinae	*Cotesia flavipes*	NC_063945
Braconidae	Microgastrinae	*Cotesia vestalis*	NC_014272
Braconidae	Opiinae	*Fopius arisanus*	MZ128286
Braconidae	Opiinae	*Psyttalia concolor*	MW279212
Braconidae	Opiinae	*Psyttalia humilis*	MW279213
Braconidae	Opiinae	*Psyttalia lounsburyi*	MW279214
Braconidae	Opiinae	*Psyttalia incisi*	OK413636
Braconidae	Opiinae	*Psyttalia carinata*	PX579120
Braconidae	Zeleinae	*Zele chlorophthalmus*	NC_039181

*Note:* *Diadegma semiclausum* and *Gotra octocincta* were used as ichneumonid outgroups; all remaining taxa belong to Braconidae.

Maximum‐likelihood analysis was performed in IQ‐TREE. Under an edge‐linked partition model, ModelFinder was used to select the optimal partitioning scheme and the best‐fitting nucleotide substitution model for each partition. Branch support was assessed using 1000 bootstrap replicates (Kalyaanamoorthy et al. 2017; Nguyen et al. 2015). Bayesian inference was conducted in MrBayes using two independent runs, each with four Markov chains for 5,000,000 generations. Trees were sampled every 1000 generations, and the first 25% of samples were discarded as burn‐in (Ronquist et al. 2012). The resulting phylogenetic trees were visualized and graphically refined using the online ChiPlot platform (Xie et al. 2023).

### 2.8 Statistical Analysis

Adult longevity was analyzed using a three‐way factorial model with temperature, diet, and sex as categorical fixed factors. Because all 720 individuals died during the observation period and longevity distributions were right‐skewed and heteroscedastic, longevity was log‐transformed before analysis. The full model included all main effects and all two‐way and three‐way interactions, and statistical inference was based on F tests using HC3 heteroscedasticity‐robust covariance estimates. Kaplan–Meier curves were retained to describe the survival patterns of the treatment groups. Arithmetic mean longevity for each treatment combination is presented as mean ± SE.

Apparent parasitism was calculated separately for the Altay and Akqi samples using the total number of puparia reared and the number confirmed to be parasitized by *P. carinata* in each region. A pooled apparent parasitism estimate was also calculated by combining data from the two regions. Morphometric measurements of parasitoids from Altay and Akqi were summarized using the mean, standard deviation, and range; no inferential tests of differences between regions were performed. All statistical analyses were conducted in OriginPro 2026, and statistical significance was defined as *p* < 0.05.

## 3 Results

### 3.1 Geographic Occurrence, Species Identification, and Host Association

*Psyttalia carinata* was detected at 12 of the 30 surveyed sites in Xinjiang (Table S1). Representative females from Altay and Akqi conformed to the species‐level diagnosis of *P. carinata* in key characters of the head, antennal flagellomeres, forewing venation, first metasomal tergite, hind leg, and ovipositor sheath. The measured ranges of the principal diagnostic characters overlapped extensively between the two populations (Table S2). Independent examination of representative specimens by Dr. Cornelis van Achterberg confirmed the species‐level identification. Review of regional taxonomic revisions, faunal checklists, taxonomic catalogs, and publicly accessible biodiversity databases yielded no reliable formal record of *P. carinata* from China. The present study therefore represents the first confirmed record of *P. carinata* from China.

Laboratory rearing and dissection provided direct evidence of the host association between *R. batava* and *P. carinata* . All examined puparia were formed by tephritid larvae that had developed within sea buckthorn fruits and subsequently exited the fruits. All adult tephritids emerging from the corresponding collection batches were identified as *R. batava* , and no other tephritid species were recovered during rearing. Adults of *P. carinata* emerged from these puparia, and both parasitoid larvae and pupae were observed within intact *R. batava* puparia. The morphology of the immature stages and of female and male adults is shown in Figure 1.

**FIGURE 1:**
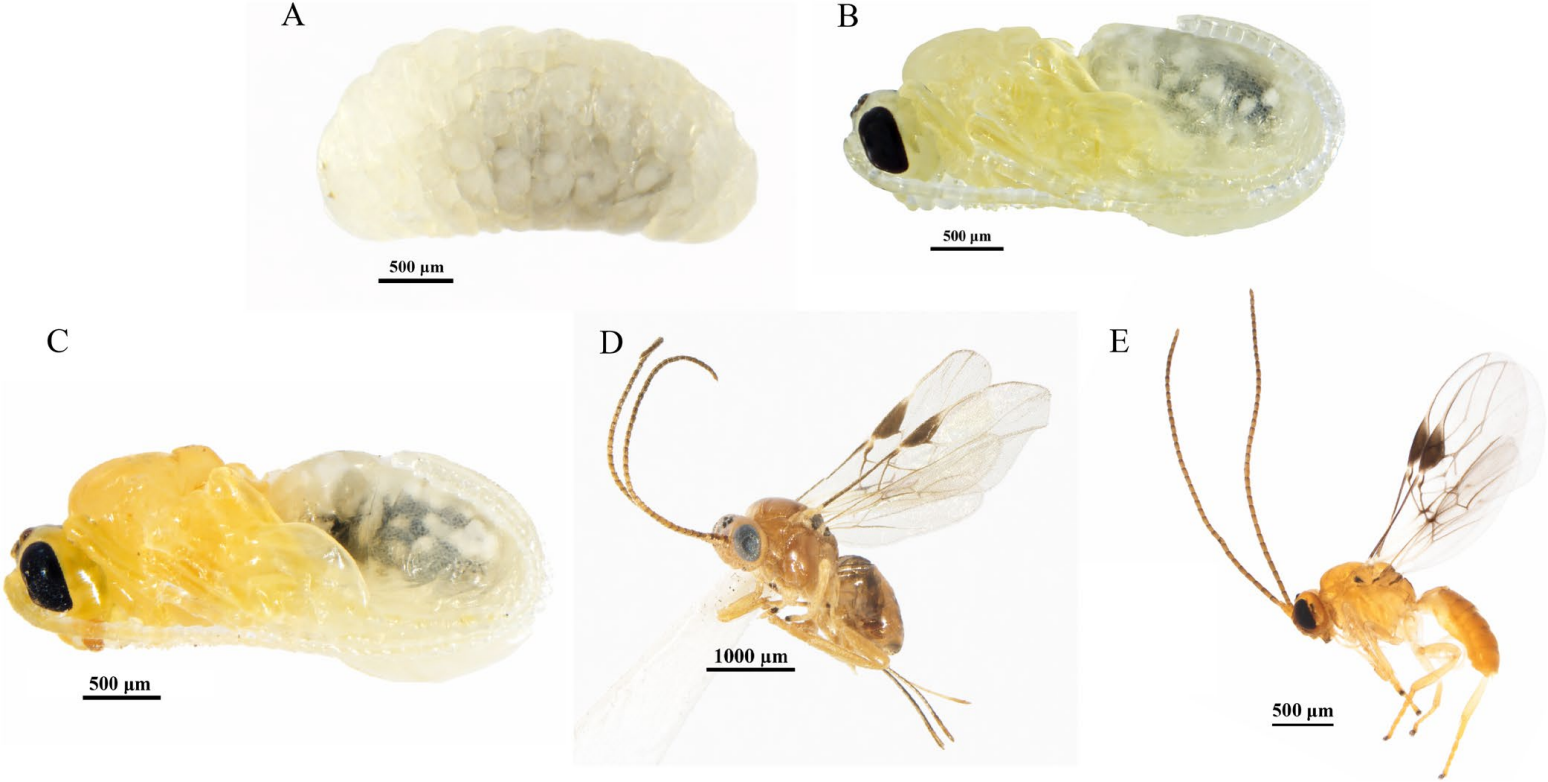
Immature stages and adults of *Psyttalia carinata*. (A) Larva of *P. carinata* inside the host puparium; (B) pre‐overwintering pupa of *P. carinata* within the host puparium; (C) post‐overwintering pupa of *P. carinata* within the host puparium; (D) adult female; (E) adult male.

### 3.2 Parasitism Biology, Overwintering Stage, and Apparent Parasitism

During the sequential dissection series, more than 900 host larvae and puparia were examined. Repeated observations of parasitoid development within the host showed that *P. carinata* develops as a solitary larval–pupal koinobiont endoparasitoid of *R. batava* . Following parasitism, host larvae continued feeding and developing within sea buckthorn fruits, subsequently exited the fruits and formed puparia. The parasitoid then completed its larval and pupal development within the host puparium. Dissections conducted before and after the overwintering period revealed *P. carinata* pupae within *R. batava* puparia, indicating that the parasitoid overwintered in the pupal stage.

From April to November in both 2024 and 2025, two Malaise traps at the fixed Altay site were sampled at 10‐day intervals. In each year, both *R. batava* and *P. carinata* exhibited a single concentrated period of adult occurrence, with the adult activity peak of *P. carinata* occurring later than that of *R. batava* . Taken together, the known univoltine life cycle of *R. batava* in the study area, pupal overwintering of the parasitoid within host puparia, and the occurrence of only one concentrated adult activity period in each monitoring year support a univoltine life cycle of *P. carinata* in the Altay study area.

Across all long‐term rearing batches, 457 adult *P. carinata* emerged. Subsequent dissection of the remaining unemerged puparia identified an additional 138 puparia containing immature parasitoids. Thus, 595 of the 8260 host puparia were classified as parasitized: 457 were represented by emerged adult parasitoids, and 138 contained parasitoid immatures detected by dissection. In the Altay samples, 409 of 5400 puparia were parasitized, corresponding to an apparent parasitism rate of 7.57%. In the Akqi samples, 186 of 2860 puparia were parasitized, corresponding to an apparent parasitism rate of 6.50%. The pooled apparent parasitism rate across both localities was 7.20%.

### 3.3 Effects of Temperature, Diet, and Sex on Adult Longevity

Temperature, diet, and sex significantly affected log‐transformed adult longevity (temperature: *F*_3,696_ = 433.01, *p* < 0.001; diet: *F*_2,696_ = 758.13, *p* < 0.001; sex: *F*_1,696_ = 171.94, *p* < 0.001). The diet × sex interaction was also significant (*F*_2,696_ = 13.51, *p* < 0.001), whereas the temperature × diet, temperature × sex, and three‐way interactions were not significant (all *p* ≥ 0.083).

Mean longevity decreased consistently with increasing temperature across all diet and sex combinations (Table 2 and Figure 2). Relative to 15°C, model‐estimated longevity declined to 63.0%, 35.2%, and 23.2% at 20°C, 25°C, and 30°C, respectively. Under the water treatment, mean female longevity decreased from 12.40 ± 0.70 days at 15°C to 2.70 ± 0.14 days at 30°C, whereas mean male longevity decreased from 10.20 ± 0.61 to 2.50 ± 0.11 days.

**TABLE 2:** Mean longevity (days) of female and male adults of *Psyttalia carinata* under different temperature and dietary treatments.

Temperature (°C)	Water		15% Honey		30% Honey	
	Female (days)	Male (days)	Female (days)	Male (days)	Female (days)	Male (days)
15	12.40 ± 0.70	10.20 ± 0.61	45.80 ± 1.96	28.40 ± 1.75	46.17 ± 2.38	29.30 ± 2.01
20	7.90 ± 0.48	6.50 ± 0.35	30.20 ± 2.08	17.60 ± 1.23	30.70 ± 2.26	18.20 ± 0.93
25	4.34 ± 0.31	3.00 ± 0.24	19.40 ± 1.23	9.95 ± 0.86	19.30 ± 1.45	11.40 ± 1.22
30	2.70 ± 0.14	2.50 ± 0.11	11.33 ± 0.83	7.20 ± 0.69	10.90 ± 0.98	7.80 ± 0.76

*Note:* Values are means ± SE; *n* = 30 adults for each temperature × diet × sex combination. Statistical analyses were based on log‐transformed longevity.

**FIGURE 2:**
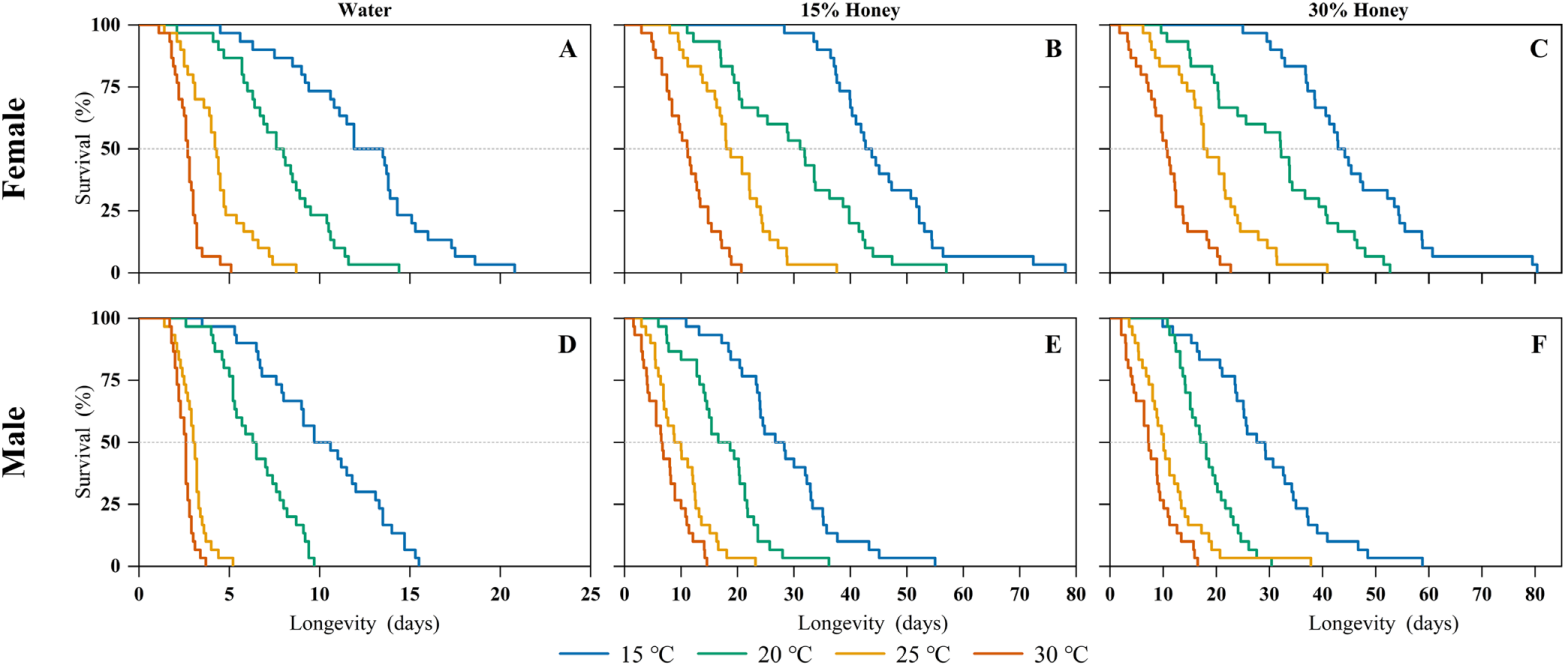
Kaplan–Meier survival curves for female and male adults of *Psyttalia carinata* under different diet and temperature treatments. Panels (A–C) show female survival under water, 15% honey, and 30% honey, respectively, whereas panels (D–F) show the corresponding male survival under the same dietary treatments. Blue, green, yellow, and orange curves represent 15°C, 20°C, 25°C, and 30°C, respectively. The horizontal dashed line indicates 50% survival.

Both honey diets markedly prolonged adult longevity relative to water, whereas mean longevity was broadly similar under the 15% and 30% honey treatments (Table 2 and Figure 2). Females generally lived longer than males, and the significant diet × sex interaction indicated that the magnitude of the sex difference varied with dietary treatment.

### 3.4 Mitochondrial Genome Organization, Structure, and Nucleotide Compositional Bias

The complete mitochondrial genome of *P. carinata* was 15,633 bp in length and was deposited in GenBank under accession number PX579120. It comprised a closed, circular, double‐stranded DNA molecule containing the typical set of 37 metazoan mitochondrial genes, including 13 protein‐coding genes, 22 tRNA genes, and two rRNA genes, together with one major non‐coding control region (Figure 3 and Table 3). Thirteen intergenic spacers and 13 gene overlaps were identified. The longest intergenic spacer, 155 bp in length, occurred between *cox2* and *trnD*, whereas the longest overlap, spanning 26 bp, was located between *trnF* and *nad5*. The control region was 809 bp long and was positioned between *trnM* and *trnQ*.

**FIGURE 3:**
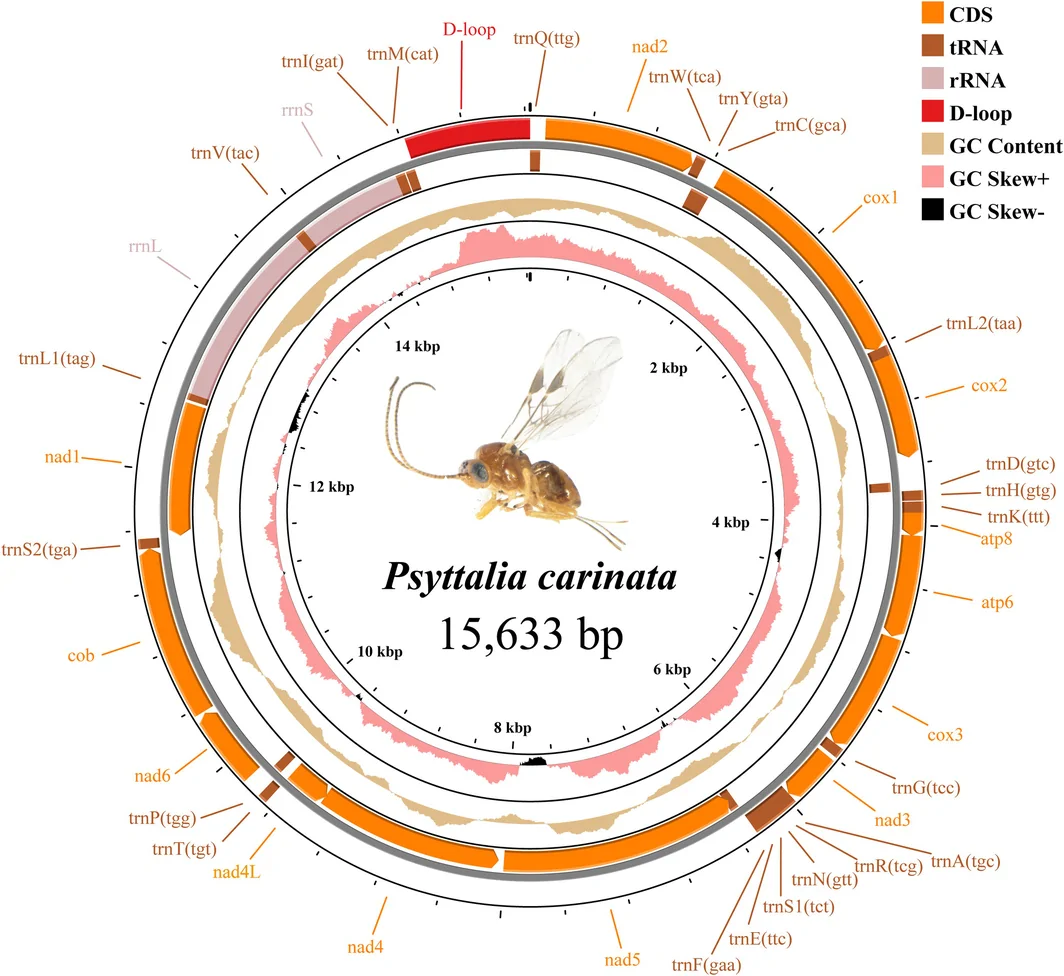
Circular map of the mitochondrial genome of *Psyttalia carinata*.

**TABLE 3:** Structure of the mitochondrial genome of *Psyttalia carinata*.

Gene	Position			Spacer/overlap (bp)	Codon		Strand
	From	To	Length (bp)		Start	Stop	
*trnQ*	1	70	70				L
*nad2*	102	1091	990	31	ATT	TAA	H
*trnW*	1090	1155	66	−2			H
*trnY*	1154	1219	66	−2			L
*trnC*	1221	1284	64	1			L
*cox1*	1285	2826	1542		ATG	TAA	H
*trnL2*	2822	2888	67	−5			H
*cox2*	2865	3557	693	−24	ATG	TAA	H
*trnD*	3713	3775	63	155			L
*trnH*	3776	3839	64				H
*trnK*	3847	3917	71	7			H
*atp8*	3918	4070	153		ATC	TAG	H
*atp6*	4067	4738	672	−4	ATA	TAG	H
*cox3*	4745	5529	785	6	ATG	TA	H
*trnG*	5529	5596	68	−1			H
*nad3*	5623	5973	351	26	ATT	TAA	H
*trnA*	5974	6034	61				H
*trnR*	6035	6101	67				H
*trnN*	6095	6159	65	−7			H
*trnS1*	6157	6224	68	−3			H
*trnE*	6224	6287	64	−1			H
*trnF*	6288	6349	62				L
*nad5*	6324	8003	1680	−26	ATT	TAA	L
*nad4*	8041	9366	1326	37	ATG	TAA	L
*nad4L*	9360	9650	291	−7	ATT	TAA	L
*trnT*	9653	9714	62	2			H
*trnP*	9715	9779	65				L
*nad6*	9841	10,344	504	61	ATA	TAA	H
*cob*	10,357	11,484	1128	12	ATG	TAA	H
*trnS2*	11,484	11,548	65	−1			H
*nad1*	11,554	12,489	936	5	ATA	TAA	L
*trnL1*	12,511	12,577	67	21			L
*rrnL*	12,558	13,877	1320	−20			L
*trnV*	13,878	13,946	69				L
*rrnS*	13,947	14,683	737				L
*trnI*	14,684	14,750	67				L
*trnM*	14,760	14,824	65	9			L
D‐loop	14,825	15,633	809				H

*Note:* Positive values indicate intergenic spacer regions, whereas negative values indicate the number of overlapping nucleotides between adjacent genes.

The mitochondrial genome exhibited a pronounced A + T bias. The proportions of A, T, G, and C were 38.5%, 44.2%, 10.3%, and 7.1%, respectively, yielding an overall A + T content of 82.7%. The control region had the highest A + T content at 91.4%, followed by the rRNA genes at 88.9% and the tRNA genes at 86.8%, whereas the protein‐coding genes had an A + T content of 80.1%. The AT‐skew values of the complete genome and the protein‐coding genes were −0.069 and −0.146, respectively, indicating an excess of T over A. By contrast, the tRNA genes, rRNA genes, and control region showed positive AT‐skew values of 0.016, 0.096, and 0.074, respectively. GC‐skew was positive for the complete genome, protein‐coding genes, tRNA genes, and control region, with values of 0.185, 0.078, 0.302, and 0.543, respectively. Only the rRNA genes exhibited a negative GC‐skew value of −0.057 (Table 4).

**TABLE 4:** Nucleotide composition of the mitochondrial genome of *Psyttalia carinata*.

Region	Length (bp)	A%	T%	C%	G%	AT (%)	GC (%)	AT‐skew	GC‐skew
Total genome	15,633	38.5	44.2	7.1	10.3	82.7	17.4	−0.069	0.185
PCGs	11,051	34.2	45.9	9.2	10.7	80.1	19.9	−0.146	0.078
tRNAs	1446	44.1	42.7	4.6	8.6	86.8	13.2	0.016	0.302
rRNAs	2057	48.7	40.2	5.9	5.3	88.9	11.2	0.096	−0.057
D‐loop	809	49.1	42.3	2.0	6.7	91.4	8.7	0.074	0.543

### 3.5 Protein‐Coding Genes, RNA Genes, and Codon Usage

The 13 protein‐coding genes had a combined length of 11,051 bp. Among them, *nad5* was the longest at 1680 bp, whereas *atp8* was the shortest at 153 bp. All protein‐coding genes initiated with canonical ATN start codons. *cox3* terminated with the incomplete stop codon TA, *atp8* and *atp6* terminated with TAG, and the remaining 10 genes terminated with TAA. Four genes, *nad5*, *nad4*, *nad4L*, and *nad1*, were encoded on the minority strand, whereas the other nine protein‐coding genes were encoded on the majority strand.

Analysis of relative synonymous codon usage revealed a pronounced bias toward codons ending in A or U, with the most frequently used codons terminating in one of these two nucleotides (Figure 4). Leucine UUR, isoleucine, phenylalanine, and methionine accounted for 14.38%, 11.60%, 10.49%, and 9.83% of the encoded amino acids, respectively, and were the most frequently represented amino acids. By contrast, cysteine and arginine were the least abundant, accounting for 0.76% and 1.17%, respectively.

**FIGURE 4:**
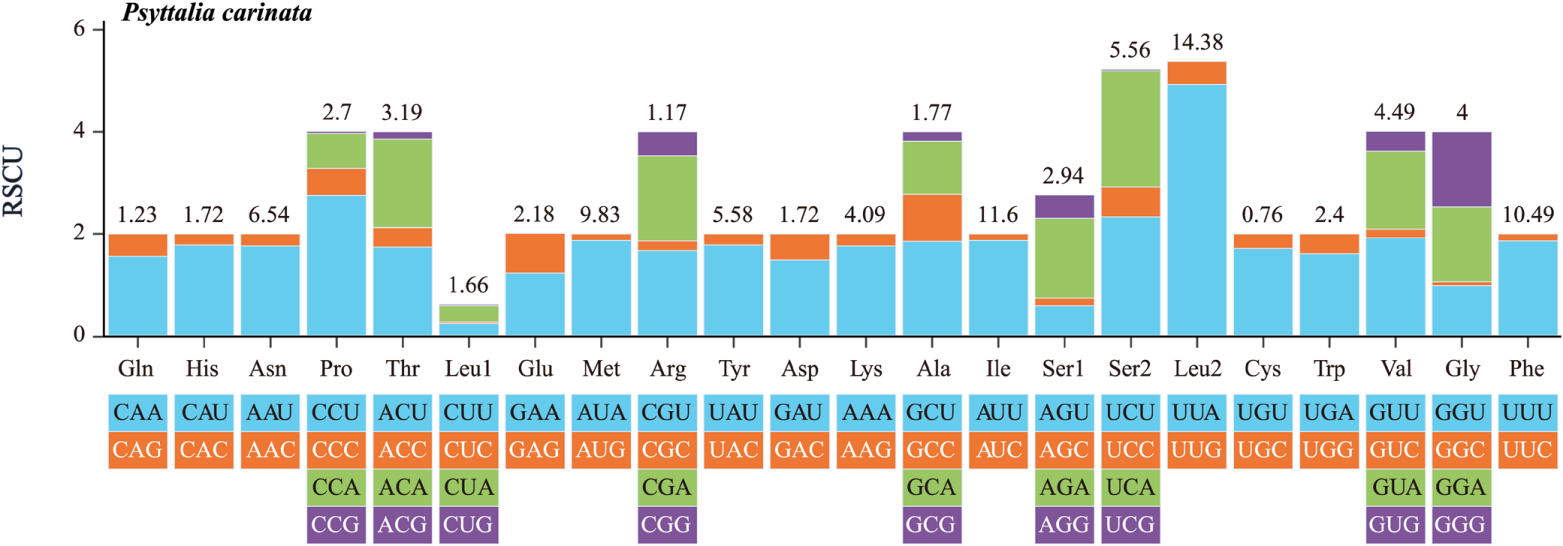
Relative synonymous codon usage and amino acid composition of the mitochondrial protein‐coding genes of *Psyttalia carinata*.

The 22 tRNA genes had a total length of 1446 bp and ranged individually from 61 to 71 bp. *trnK* was the longest at 71 bp, whereas *trnA* was the shortest at 61 bp. All tRNAs except *trnS1* could be folded into the typical cloverleaf secondary structure; *trnS1* lacked a complete dihydrouridine arm (Figure 5). Both rRNA genes were encoded on the minority strand. The large ribosomal RNA gene, *rrnL*, was 1320 bp long and located between *trnL1* and *trnV*, whereas the small ribosomal RNA gene, *rrnS*, was 737 bp long and located between *trnV* and *trnI*.

**FIGURE 5:**
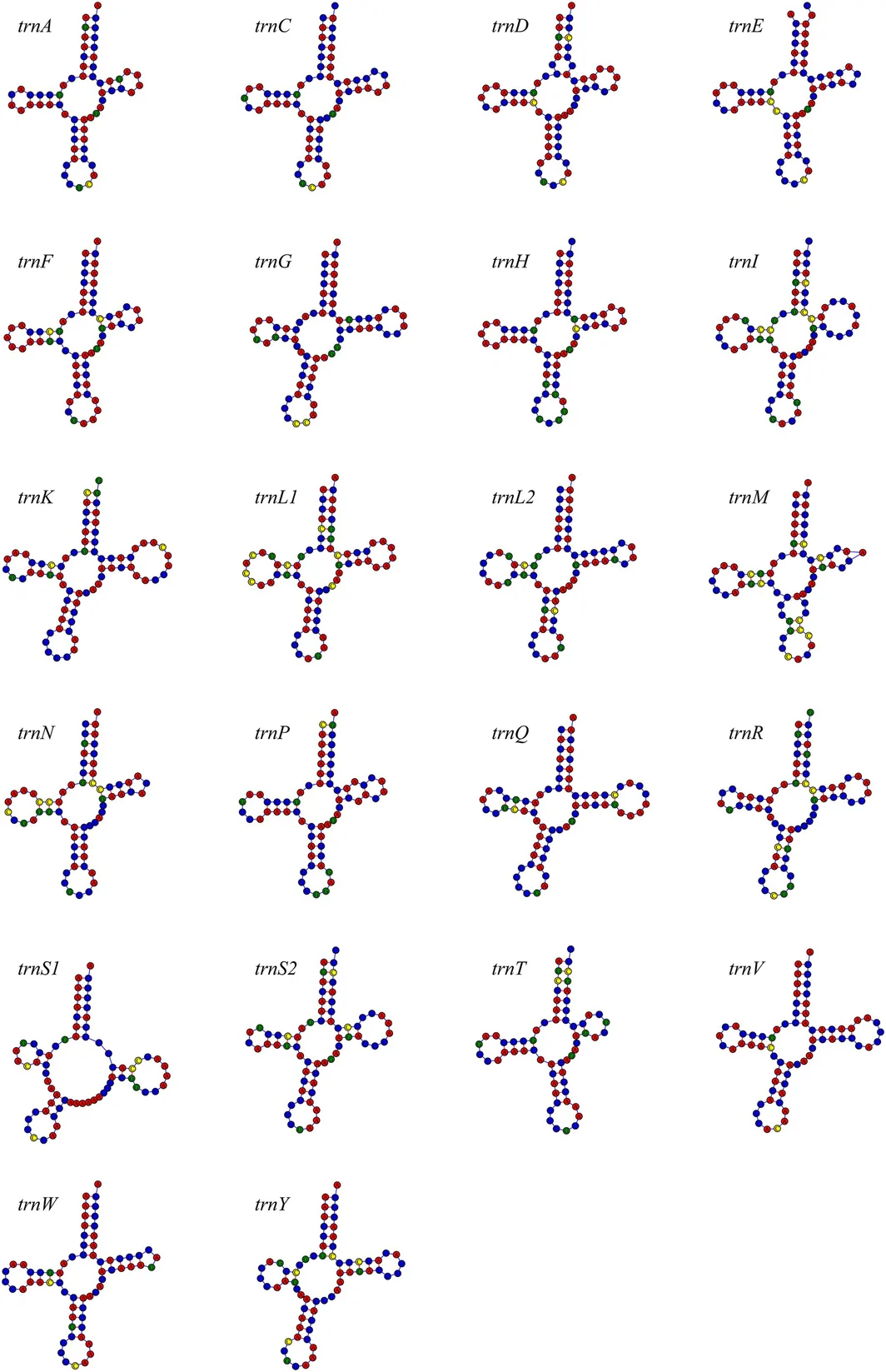
Secondary structures of tRNAs in the mitochondrial genome of *Psyttalia carinata*.

### 3.6 Mitochondrial Gene Order and Rearrangements

Gene‐order comparisons among the five available *Psyttalia* mitogenome assemblies showed that the relative positions and transcriptional orientations of the 13 protein‐coding genes and two rRNA genes were conserved across the homologous regions examined, whereas the observed intrageneric variation involved tRNA genes (Figure 6). Relative to the reference arrangements of *Daphnia pulex* and *Formica fusca* , the compared *Psyttalia* mitogenomes shared several tRNA rearrangements, particularly in the regions surrounding cox2–atp8 and nad2–cox1, with additional rearrangement of the rrnS–nad2 interval evident in the complete mitogenomes. No translocations of protein‐coding or rRNA genes were detected in the regions available for direct comparison.

**FIGURE 6:**
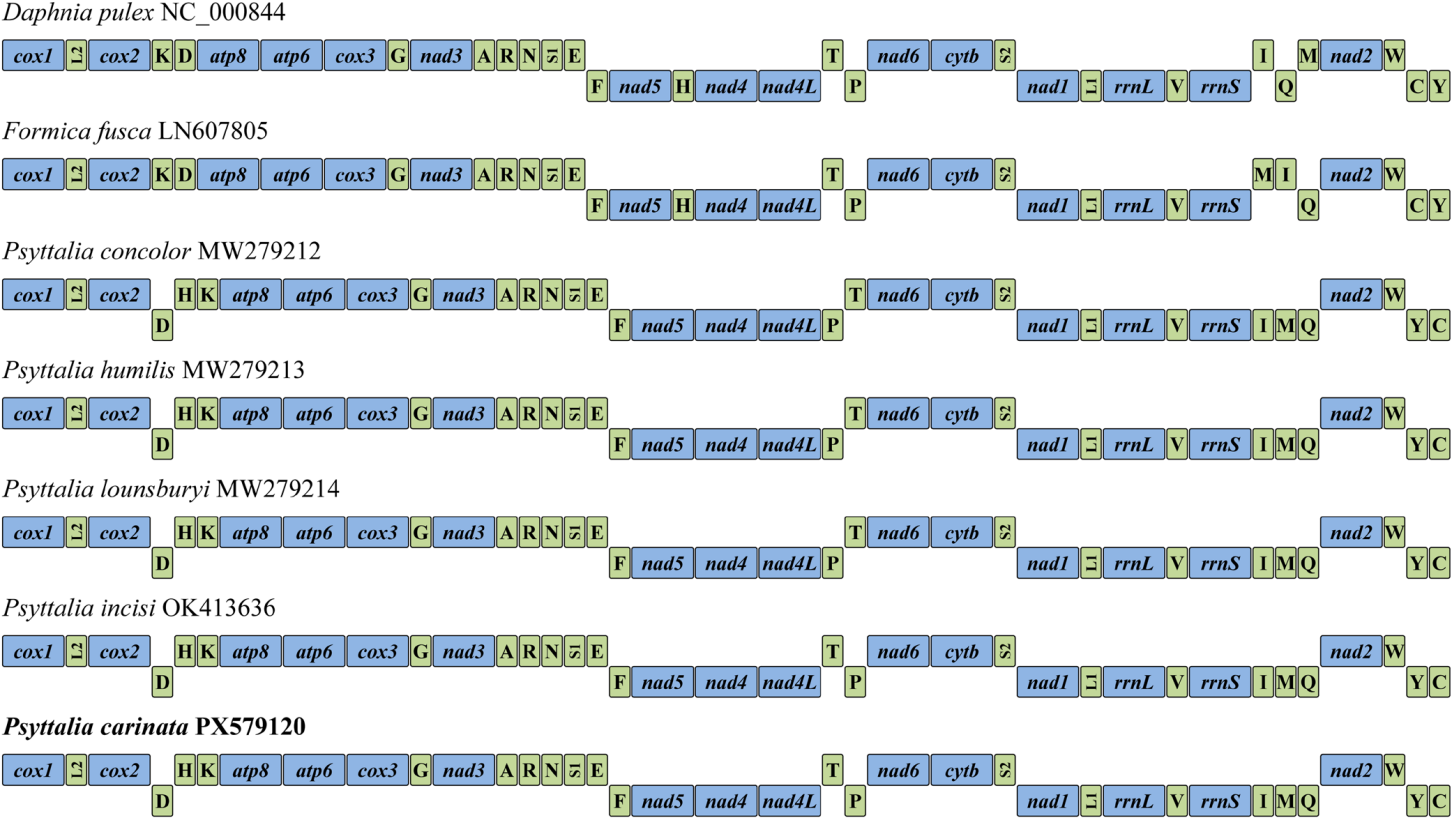
Comparison of mitochondrial gene arrangements among five *Psyttalia* species and two reference taxa.

Under the current taxon sampling, all five *Psyttalia* species shared the cox2–trnD–trnH–trnK–atp8 and nad2–trnW–trnY–trnC–cox1 arrangements. The rrnS–trnI–trnM–trnQ–nad2 arrangement was recovered in the complete mitogenomes of *P. carinata* , *P. incisi*, and *P. concolor* , whereas the corresponding A + T‐rich‐region–trnQ interval was not fully recovered in *P. humilis* and *P. lounsburyi*. Together, these conserved gene clusters represent relatively stable gene‐order features within the currently available *Psyttalia* mitogenome data.

Intrageneric variation was concentrated in the order of trnT and trnP between nad4L and nad6. Both *P. carinata* and *P. incisi* exhibited the nad4L–trnT–trnP–nad6 arrangement and shared the same complete mitochondrial gene order. In contrast, *P. concolor* , *P. humilis* , and *P. lounsburyi* shared the nad4L–trnP–trnT–nad6 arrangement. The five sampled *Psyttalia* species therefore represented two local gene‐order types in this region: the trnT–trnP type and the trnP–trnT type. No additional differences were detected among the homologous regions available for direct comparison.

### 3.7 Phylogenetic Relationships

Bayesian inference and maximum‐likelihood analyses based on all three codon positions of the 13 mitochondrial protein‐coding genes recovered identical topologies for relationships within *Psyttalia* (Figure 7). The five sampled *Psyttalia* species formed a monophyletic group with strong nodal support (BI: PP = 1.00; ML: BS = 92). Within the genus, *P. concolor* and *P. humilis* were recovered as sister species with maximal support, and this clade was subsequently grouped with *P. lounsburyi*. *P. carinata* was recovered as the sister lineage to the clade comprising these three species, with strong support in both analyses (PP = 0.97; BS = 100). *Psyttalia incisi* was placed as the sister lineage to the clade containing the other four *Psyttalia* species. The monophyletic *Psyttalia* clade was further recovered as the sister group to *Fopius arisanus*, again with strong support.

**FIGURE 7:**
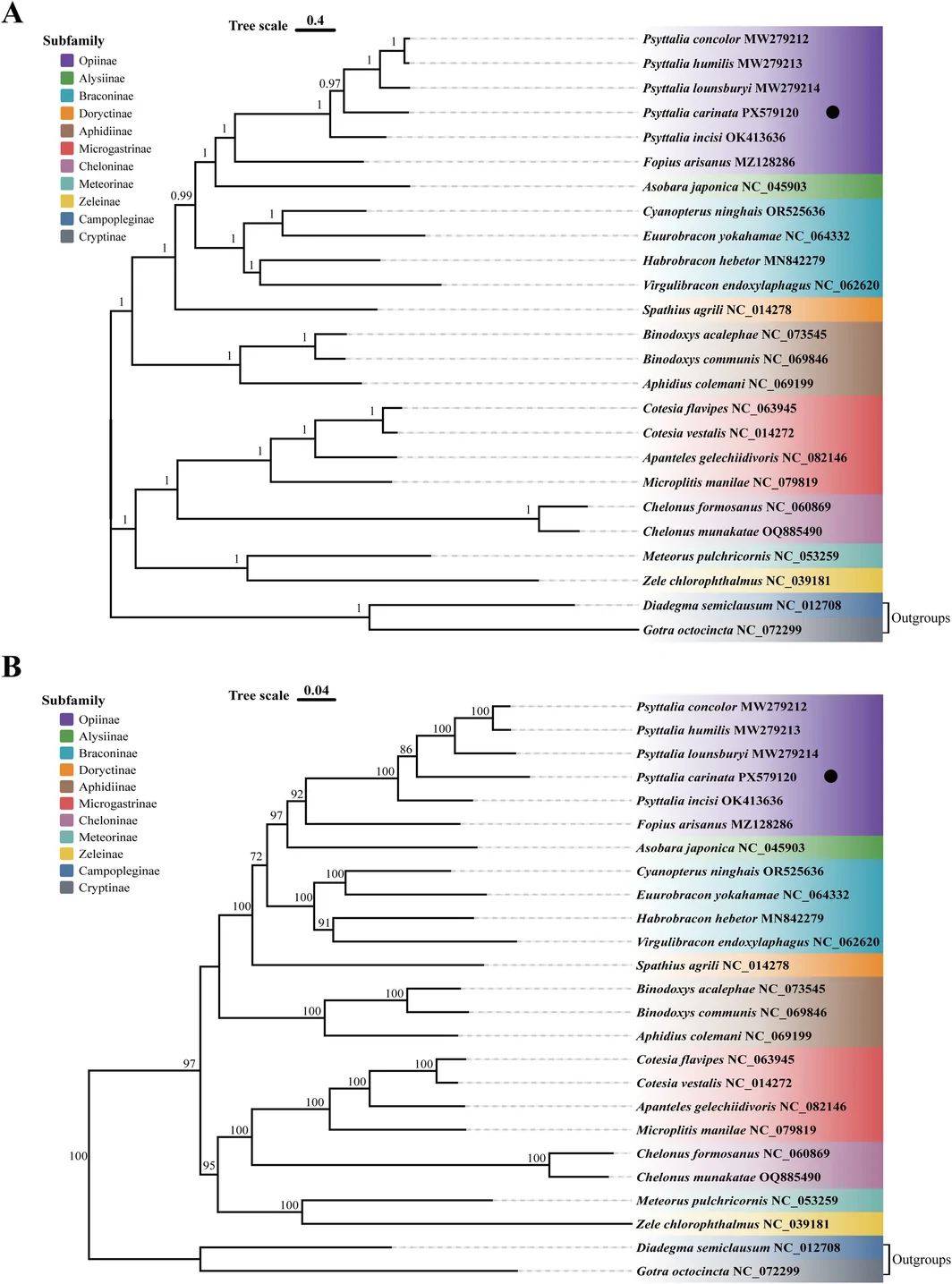
Phylogenetic position of *Psyttalia carinata* within Braconidae inferred from mitochondrial protein‐coding genes (PCGs). (A) Bayesian inference (BI) tree; (B) maximum‐likelihood (ML) tree.

Overall, both analytical approaches consistently supported the generic placement of *P. carinata* and indicated that it is most closely related to the clade comprising *P. concolor* , *P. humilis* , and *P. lounsburyi*.

## 4 Discussion

The principal contribution of this study lies not only in providing the first confirmed record of *P. carinata* from China, but also in integrating geographic, biological, and molecular evidence to clarify its ecological significance and systematic placement. For opiine parasitoids associated with fruit‐infesting tephritids, the value of a distribution record often depends on whether it is accompanied by unambiguous host information and verifiable life history evidence, because such information directly informs the reconstruction of regional natural enemy networks and the assessment of potential biological control functions. Recent reviews have emphasized that the application potential of fruit fly parasitoids in agroecosystems is closely related to host range, regional establishment capacity, and persistence under field conditions, and that the identification and conservation of indigenous parasitoid resources are particularly important in arid and semiarid agricultural systems (Garcia et al. 2020; Saabna and Keasar 2024). In the present study, species identity was established through morphological diagnosis and expert verification, whereas direct host association was demonstrated through parasitoid emergence from *R. batava* puparia of confirmed origin and observations of immature parasitoids within host puparia. By linking the new Chinese distribution record of *P. carinata* directly to its parasitic use of *R. batava* , this study extends the finding beyond the addition of a geographic locality and gives it clearer biogeographic and ecological significance.

The developmental pattern observed in *P. carinata* is consistent with the larval–pupal koinobiont strategy reported for other parasitoid Hymenoptera, in which the parasitized host continues development before the parasitoid completes its immature development within the host puparium (Pennacchio and Strand 2006). Together with pupal overwintering and the single concentrated annual period of adult activity recorded across 2 years, this pattern supports a univoltine life cycle of *P. carinata* in the Altay population. Importantly, the later activity peak of *P. carinata* relative to adult *R. batava* should not necessarily be interpreted as a phenological mismatch. Adult fly emergence and oviposition precede the development of larval stages available for parasitism, so the observed temporal offset is consistent with parasitoid activity tracking the availability of suitable host stages (Furlong and Zalucki 2017; Ramos Aguila et al. 2023). Of the 8260 host puparia examined, 595 were confirmed to be parasitized by *P. carinata* , yielding a pooled apparent parasitism rate of 7.20%. This result indicates that *P. carinata* is a regularly occurring natural enemy in the local *R. batava* system and provides a quantitative baseline for evaluating its ecological role.

The adult longevity experiment showed that temperature, diet, and sex significantly affected the longevity of *P. carinata* , and that the magnitude of the sex difference varied among dietary treatments. Longevity declined markedly across all diet‐by‐sex combinations as temperature increased from 15°C to 30°C, suggesting that high temperatures may reduce the time available for somatic maintenance, mating, and host searching. This trend is broadly consistent with previous evidence that elevated temperatures accelerate metabolic expenditure and can reduce parasitoid survival and ecological performance (Furlong and Zalucki 2017; Hance et al. 2007; Malinski et al. 2024; Spahn and Lill 2022). Relative to water alone, both 15% and 30% honey solutions markedly prolonged the longevity of females and males, in agreement with studies showing that carbohydrate resources replenish parasitoid energy reserves, extend adult lifespan, and support potential reproductive activity (Fernández de Bobadilla et al. 2024; Kishinevsky and Ives 2024; Lee 2024; Lee and Heimpel 2008; Urbaneja‐Bernat et al. 2023). Mean longevity was similar under the two honey concentrations, suggesting that increasing honey concentration from 15% to 30% provided little additional longevity benefit under the present laboratory conditions. The significant diet × sex interaction further showed that the magnitude of the female longevity advantage depended on nutritional treatment, with a larger proportional difference under the honey diets than under water alone. Collectively, these results define the direction and magnitude of adult longevity responses to temperature and sugar availability and suggest that access to carbohydrate resources may contribute to the persistence of *P. carinata* in arid habitats. Future experiments incorporating fluctuating temperatures, fecundity, and host searching behavior will help determine how these longevity responses translate into field performance.

The newly obtained mitochondrial genome of *P. carinata* contained the typical complement of 37 insect mitochondrial genes and exhibited a pronounced A + T bias and preferential use of codons ending in A or U, consistent with the general compositional patterns reported for insect and braconid mitogenomes (Cameron 2014, 2025; Jasso‐Martínez et al. 2022; Wei et al. 2010). The principal molecular contribution of this study is the addition of a complete, circularized mitochondrial genome of *P. carinata* from a reliably identified specimen and its comparison with the four other available *Psyttalia* mitogenome assemblies (Powell et al. 2020; Yang et al. 2022). Across the homologous regions examined, the relative positions and transcriptional orientations of protein‐coding and rRNA genes were conserved, whereas the observed intrageneric variation involved tRNA genes. This pattern is consistent with the predominance of tRNA translocations, inversions, and localized gene‐cluster rearrangements reported across Hymenoptera and Braconidae (Cameron 2025; Dowton et al. 2009; Wei et al. 2010). All five species shared the cox2–trnD–trnH–trnK–atp8 and nad2–trnW–trnY–trnC–cox1 arrangements, whereas two alternative arrangements occurred in the nad4L–nad6 region. *P. carinata* and *P. incisi* possessed the trnT–trnP arrangement, while *P. concolor* , *P. humilis* , and *P. lounsburyi* possessed trnP–trnT. These local arrangements provide candidate structural characters for examining mitochondrial gene‐order evolution within the genus, although their evolutionary direction and broader taxonomic informativeness require denser intrageneric sampling (Dowton and Austin 1999; Dowton et al. 2009).

The contrasting signals provided by gene order and phylogeny inferred from sequence data offer a complementary perspective on mitochondrial genome evolution within *Psyttalia*. Both Bayesian and maximum‐likelihood analyses supported the monophyly of the sampled *Psyttalia* species and placed *P. carinata* as sister to the clade comprising *P. concolor* , *P. humilis* , and *P. lounsburyi*, whereas *P. incisi* was recovered as sister to the clade containing the other four species. Nevertheless, *P. carinata* and *P. incisi* shared the same gene order across their complete mitogenomes despite not being recovered as sister species. Conversely, *P. carinata* was most closely related in the phylogeny inferred from sequence data to a clade characterized by the alternative trnP–trnT arrangement. This incongruence indicates that local similarity in gene order does not necessarily correspond to the closest sequence‐based phylogenetic relationship and may reflect the retention of an earlier arrangement, lineage‐specific rearrangement, or homoplastic change. Integrating gene‐order information with phylogeny inferred from sequence data therefore provides a useful framework for evaluating alternative scenarios of mitochondrial genome evolution (Cameron 2014, 2025; Jasso‐Martínez et al. 2022; Mao et al. 2015). Because intrageneric sampling remains limited, the direction and number of rearrangement events cannot yet be resolved. Broader species sampling, together with independent nuclear markers, will be required to evaluate these evolutionary patterns and assess the stability of the current phylogenetic topology.

Overall, morphological diagnosis and expert verification confirm *P. carinata* as a new record for China, while emergence from puparia of known origin and observations of immature stages establish *R. batava* as a verified host. The study further documents its solitary koinobiont development, pupal overwintering, apparent parasitism, and responses of adult longevity to temperature, diet, and sex. A single concentrated annual activity period was recorded in the monitored Altay population, supporting a univoltine life cycle at that site. The complete mitochondrial genome expands molecular resources for *Psyttalia* and shows that variation among the five available assemblies is concentrated mainly in tRNA gene order. The discordance between the shared *trnT–trnP* arrangement of *P. carinata* and *P. incisi* and their positions in the sequence phylogeny indicates that local similarity in gene order does not necessarily identify the closest relationship. These findings establish a biological and molecular reference for *P. carinata* and support its consideration as a regional natural enemy of *R. batava* . Standardized field studies across additional regions and years, together with data on fecundity, host searching, and nuclear markers, will be needed to evaluate its contribution to biological control and refine relationships within *Psyttalia*.

## Author Contributions

**Kailiang Li:** data curation (equal), formal analysis (equal), investigation (equal), methodology (equal), software (equal), visualization (equal), writing – original draft (equal). **Zhiqiang Ge:** data curation (equal), formal analysis (equal), visualization (equal). **Zhenyu Zhang:** investigation (equal), methodology (equal). **Wenchao Guo:** methodology (equal), project administration (equal). **Hongying Hu:** funding acquisition (lead), project administration (equal), writing – review and editing (lead).

## Funding

This study was supported by the Major Science and Technology Projects of Xinjiang Uygur Autonomous Region (2023A02006).

## Conflicts of Interest

The authors declare no conflicts of interest.

## Supporting information


**Table S1:** Geographic information and detection status of the sea buckthorn fruit fly *Rhagoletis batava* and its larval parasitoid *Psyttalia carinata* at survey sites across Xinjiang, China.

**Table S2a:** Morphometric measurements of representative female *Psyttalia carinata* specimens from Altay and Aheqi, Xinjiang, China.
**Table S2b:** Comparison of diagnostic morphometric ratios in female *Psyttalia carinata*.

## Data Availability

The complete mitochondrial genome sequence of *Psyttalia carinata* generated in this study has been deposited in GenBank under accession number PX579120. Geographic information and detection records from the regional survey are provided in Table S1, and morphometric measurements of representative female *P. carinata* specimens are provided in Table S2.

## References

[ece374415-bib-0001] Abram, P. K. , J. Brodeur , A. Urbaneja , and A. Tena . 2019. “Nonreproductive Effects of Insect Parasitoids on Their Hosts.” Annual Review of Entomology 64: 259–276. 10.1146/annurev-ento-011118-111753. 30312554

[ece374415-bib-0002] Aleknavičius, D. , and V. Būda . 2019. “Trapping Peculiarities, Flight and Mating Dynamics of Sea Buckthorn Fruit Fly ( *Rhagoletis batava* ) in Lithuania.” Zemdirbyste‐Agriculture 106, no. 1: 81–86. 10.13080/z-a.2019.106.011.

[ece374415-bib-0003] Bernt, M. , A. Donath , F. Jühling , et al. 2013. “MITOS: Improved De Novo Metazoan Mitochondrial Genome Annotation.” Molecular Phylogenetics and Evolution 69, no. 2: 313–319. 10.1016/j.ympev.2012.08.023. 22982435

[ece374415-bib-0004] Blažytė‐Čereškienė, L. , V. Būda , V. Apšegaitė , et al. 2022. “Sea Buckthorn *Hippophae rhamnoides* and Fruit Flies *Rhagoletis batava* : Search for Volatile Semiochemicals Involved in Pest Attraction.” Horticulturae 8, no. 2: 179. 10.3390/horticulturae8020179.

[ece374415-bib-0005] Cameron, S. L. 2014. “Insect Mitochondrial Genomics: Implications for Evolution and Phylogeny.” Annual Review of Entomology 59: 95–117. 10.1146/annurev-ento-011613-162007. 24160435

[ece374415-bib-0006] Cameron, S. L. 2025. “Insect Mitochondrial Genomics: A Decade of Progress.” Annual Review of Entomology 70: 83–101. 10.1146/annurev-ento-013024-015553. 39259965

[ece374415-bib-0007] Capella‐Gutiérrez, S. , J. M. Silla‐Martínez , and T. Gabaldón . 2009. “trimAl: A Tool for Automated Alignment Trimming in Large‐Scale Phylogenetic Analyses.” Bioinformatics 25, no. 15: 1972–1973. 10.1093/bioinformatics/btp348. PMC2712344 19505945

[ece374415-bib-0008] Carmichael, A. E. , R. A. Wharton , and A. R. Clarke . 2005. “Opiine Parasitoids (Hymenoptera: Braconidae) of Tropical Fruit Flies (Diptera: Tephritidae) of the Australian and South Pacific Region.” Bulletin of Entomological Research 95, no. 6: 545–569. 10.1079/BER2005383. 16336703

[ece374415-bib-0009] Castro, L. R. , A. D. Austin , and M. Dowton . 2002. “Contrasting Rates of Mitochondrial Molecular Evolution in Parasitic Diptera and Hymenoptera.” Molecular Biology and Evolution 19, no. 7: 1100–1113. 10.1093/oxfordjournals.molbev.a004168. 12082129

[ece374415-bib-0010] Chan, P. P. , B. Y. Lin , A. J. Mak , and T. M. Lowe . 2021. “tRNAscan‐SE 2.0: Improved Detection and Functional Classification of Transfer RNA Genes.” Nucleic Acids Research 49, no. 16: 9077–9096. 10.1093/nar/gkab688. PMC8450103 34417604

[ece374415-bib-0011] Chen, S. , Y. Zhou , Y. Chen , and J. Gu . 2018. “Fastp: An Ultra‐Fast All‐in‐One FASTQ Preprocessor.” Bioinformatics 34, no. 17: i884–i890. 10.1093/bioinformatics/bty560. PMC6129281 30423086

[ece374415-bib-0012] Donath, A. , F. Jühling , M. Al‐Arab , et al. 2019. “Improved Annotation of Protein‐Coding Genes Boundaries in Metazoan Mitochondrial Genomes.” Nucleic Acids Research 47, no. 20: 10543–10552. 10.1093/nar/gkz833. PMC6847864 31584075

[ece374415-bib-0013] Dowton, M. , and A. D. Austin . 1999. “Evolutionary Dynamics of a Mitochondrial Rearrangement “Hot Spot” in the Hymenoptera.” Molecular Biology and Evolution 16, no. 2: 298–309. 10.1093/oxfordjournals.molbev.a026111. 10028295

[ece374415-bib-0014] Dowton, M. , S. L. Cameron , J. I. Dowavic , A. D. Austin , and M. F. Whiting . 2009. “Characterization of 67 Mitochondrial tRNA Gene Rearrangements in the Hymenoptera Suggests That Mitochondrial tRNA Gene Position Is Selectively Neutral.” Molecular Biology and Evolution 26, no. 7: 1607–1617. 10.1093/molbev/msp072. 19359443

[ece374415-bib-0015] Dowton, M. , L. R. Castro , S. L. Campbell , S. D. Bargon , and A. D. Austin . 2003. “Frequent Mitochondrial Gene Rearrangements at the Hymenopteran nad3–nad5 Junction.” Journal of Molecular Evolution 56, no. 5: 517–526. 10.1007/s00239-002-2420-3. 12698290

[ece374415-bib-0016] Fernández de Bobadilla, M. , N. M. Ramírez , M. Calvo‐Agudo , M. Dicke , and A. Tena . 2024. “Honeydew Management to Promote Biological Control.” Current Opinion in Insect Science 61: 101151. 10.1016/j.cois.2023.101151. 38097038

[ece374415-bib-0017] Furlong, M. J. , and M. P. Zalucki . 2017. “Climate Change and Biological Control: The Consequences of Increasing Temperatures on Host–Parasitoid Interactions.” Current Opinion in Insect Science 20: 39–44. 10.1016/j.cois.2017.03.006. 28602234

[ece374415-bib-0018] Garcia, F. R. M. , S. M. Ovruski , L. Suárez , J. Cancino , and O. E. Liburd . 2020. “Biological Control of Tephritid Fruit Flies in the Americas and Hawaii: A Review of the Use of Parasitoids and Predators.” Insects 11, no. 10: 662. 10.3390/insects11100662. PMC7600837 32993000

[ece374415-bib-0019] Grant, J. R. , E. Enns , E. Marinier , et al. 2023. “Proksee: In‐Depth Characterization and Visualization of Bacterial Genomes.” Nucleic Acids Research 51, no. W1: W484–W492. 10.1093/nar/gkad326. PMC10320063 37140037

[ece374415-bib-0020] Hance, T. , J. van Baaren , P. Vernon , and G. Boivin . 2007. “Impact of Extreme Temperatures on Parasitoids in a Climate Change Perspective.” Annual Review of Entomology 52: 107–126. 10.1146/annurev.ento.52.110405.091333. 16846383

[ece374415-bib-0021] Hawkins, B. A. 1994. Pattern and Process in Host–Parasitoid Interactions. Cambridge University Press. 10.1017/CBO9780511721885.

[ece374415-bib-0022] Heimpel, G. E. , P. K. Abram , and J. Brodeur . 2021. “A Phylogenetic Perspective on Parasitoid Host Ranges With Implications for Biological Control.” Current Opinion in Insect Science 44: 95–100. 10.1016/j.cois.2021.04.003. 33901732

[ece374415-bib-0023] Jasso‐Martínez, J. M. , D. L. J. Quicke , S. A. Belokobylskij , et al. 2022. “Mitochondrial Phylogenomics and Mitogenome Organization in the Parasitoid Wasp Family Braconidae (Hymenoptera: Ichneumonoidea).” BMC Ecology and Evolution 22: 46. 10.1186/s12862-022-01983-1. PMC9006417 35413835

[ece374415-bib-0024] Kalyaanamoorthy, S. , B. Q. Minh , T. K. F. Wong , A. von Haeseler , and L. S. Jermiin . 2017. “ModelFinder: Fast Model Selection for Accurate Phylogenetic Estimates.” Nature Methods 14, no. 6: 587–589. 10.1038/nmeth.4285. PMC5453245 28481363

[ece374415-bib-0025] Katoh, K. , and D. M. Standley . 2013. “MAFFT Multiple Sequence Alignment Software Version 7: Improvements in Performance and Usability.” Molecular Biology and Evolution 30, no. 4: 772–780. 10.1093/molbev/mst010. PMC3603318 23329690

[ece374415-bib-0026] Kishinevsky, M. , and A. R. Ives . 2024. “Longevity of Hymenopteran Parasitoids in Natural Versus Agricultural Habitats and Implications for Biological Control.” Ecological Applications 34, no. 6: e3009. 10.1002/eap.3009. 38978401

[ece374415-bib-0027] Kück, P. , and G. C. Longo . 2014. “FASconCAT‐G: Extensive Functions for Multiple Sequence Alignment Preparations Concerning Phylogenetic Studies.” Frontiers in Zoology 11: 81. 10.1186/s12983-014-0081-x. PMC4243772 25426157

[ece374415-bib-0028] Kumar, S. , G. Stecher , M. Suleski , M. Sanderford , S. Sharma , and K. Tamura . 2024. “MEGA12: Molecular Evolutionary Genetic Analysis Version 12 for Adaptive and Green Computing.” Molecular Biology and Evolution 41, no. 12: msae263. 10.1093/molbev/msae263. PMC11683415 39708372

[ece374415-bib-0029] Lee, J. C. 2024. “Flourishing With Sugars—Following the Fate of Parasitoids in the Field.” Current Opinion in Insect Science 61: 101158. 10.1016/j.cois.2023.101158. 38184071

[ece374415-bib-0030] Lee, J. C. , and G. E. Heimpel . 2008. “Floral Resources Impact Longevity and Oviposition Rate of a Parasitoid in the Field.” Journal of Animal Ecology 77, no. 3: 565–572. 10.1111/j.1365-2656.2008.01355.x. 18248386

[ece374415-bib-0031] Li, X. , C. van Achterberg , and J. Tan . 2013. “Revision of the Subfamily Opiinae (Hymenoptera, Braconidae) From Hunan (China), Including Thirty‐Six New Species and Two New Genera.” ZooKeys 268: 1–186. 10.3897/zookeys.268.4071. PMC3592199 23653521

[ece374415-bib-0032] Ma, Q. , N.‐X. He , H. Huang , et al. 2023. “ *Hippophae rhamnoides* L.: A Comprehensive Review on the Botany, Traditional Uses, Phytonutrients, Health Benefits, Quality Markers, and Applications.” Journal of Agricultural and Food Chemistry 71, no. 12: 4769–4788. 10.1021/acs.jafc.2c06916. 36930583

[ece374415-bib-0033] Malinski, K. H. , M. E. Moore , and J. G. Kingsolver . 2024. “Heat Stress and Host–Parasitoid Interactions: Lessons and Opportunities in a Changing Climate.” Current Opinion in Insect Science 64: 101225. 10.1016/j.cois.2024.101225. 38936473

[ece374415-bib-0034] Mao, M. , T. Gibson , and M. Dowton . 2015. “Higher‐Level Phylogeny of the Hymenoptera Inferred From Mitochondrial Genomes.” Molecular Phylogenetics and Evolution 84: 34–43. 10.1016/j.ympev.2014.12.009. 25542648

[ece374415-bib-0035] Meng, G. , Y. Li , C. Yang , and S. Liu . 2019. “MitoZ: A Toolkit for Animal Mitochondrial Genome Assembly, Annotation and Visualization.” Nucleic Acids Research 47, no. 11: e63. 10.1093/nar/gkz173. PMC6582343 30864657

[ece374415-bib-0036] Nguyen, L.‐T. , H. A. Schmidt , A. von Haeseler , and B. Q. Minh . 2015. “IQ‐TREE: A Fast and Effective Stochastic Algorithm for Estimating Maximum‐Likelihood Phylogenies.” Molecular Biology and Evolution 32, no. 1: 268–274. 10.1093/molbev/msu300. PMC4271533 25371430

[ece374415-bib-0037] Pennacchio, F. , and M. R. Strand . 2006. “Evolution of Developmental Strategies in Parasitic Hymenoptera.” Annual Review of Entomology 51: 233–258. 10.1146/annurev.ento.51.110104.151029. 16332211

[ece374415-bib-0038] Perna, N. T. , and T. D. Kocher . 1995. “Patterns of Nucleotide Composition at Fourfold Degenerate Sites of Animal Mitochondrial Genomes.” Journal of Molecular Evolution 41, no. 3: 353–358. 10.1007/BF00186547. 7563121

[ece374415-bib-0039] Powell, C. , V. Caleca , C. Rhode , L. Teixeira da Costa , and B. van Asch . 2020. “New Mitochondrial Gene Rearrangement in *Psyttalia concolor*, *P. humilis* and *P. lounsburyi* (Hymenoptera: Braconidae), Three Parasitoid Species of Economic Interest.” Insects 11, no. 12: 854. 10.3390/insects11120854. PMC7761351 33276418

[ece374415-bib-0040] Quicke, D. L. J. 2014. The Braconid and Ichneumonid Parasitoid Wasps: Biology, Systematics, Evolution and Ecology. Wiley Blackwell. 10.1002/9781118907085.

[ece374415-bib-0041] Ramos Aguila, L. C. , X. Li , K. S. Akutse , et al. 2023. “Host–Parasitoid Phenology, Distribution, and Biological Control Under Climate Change.” Life 13, no. 12: 2290. 10.3390/life13122290. PMC10744521 38137891

[ece374415-bib-0042] Ranwez, V. , S. Harispe , F. Delsuc , and E. J. P. Douzery . 2011. “MACSE: Multiple Alignment of Coding Sequences Accounting for Frameshifts and Stop Codons.” PLoS One 6, no. 9: e22594. 10.1371/journal.pone.0022594. PMC3174933 21949676

[ece374415-bib-0043] Ronquist, F. , M. Teslenko , P. van der Mark , et al. 2012. “MrBayes 3.2: Efficient Bayesian Phylogenetic Inference and Model Choice Across a Large Model Space.” Systematic Biology 61, no. 3: 539–542. 10.1093/sysbio/sys029. PMC3329765 22357727

[ece374415-bib-0044] Saabna, N. , and T. Keasar . 2024. “Parasitoids for Biological Control in Dryland Agroecosystems.” Current Opinion in Insect Science 64: 101226. 10.1016/j.cois.2024.101226. 38944273

[ece374415-bib-0045] Seehausen, M. L. , N. Ris , L. Driss , et al. 2020. “Evidence for a Cryptic Parasitoid Species Reveals Its Suitability as a Biological Control Agent.” Scientific Reports 10, no. 1: 19096. 10.1038/s41598-020-76180-5. PMC7645786 33154398

[ece374415-bib-0046] Spahn, R. , and J. T. Lill . 2022. “Higher Temperatures Reduce the Efficacy of a Key Biocontrol Parasitoid.” Biological Control 176: 105079. 10.1016/j.biocontrol.2022.105079.

[ece374415-bib-0047] Tang, P. , F. Zhu , C. Zheng , et al. 2019. “Mitochondrial Phylogenomics of the Hymenoptera.” Molecular Phylogenetics and Evolution 131: 8–18. 10.1016/j.ympev.2018.10.040. 30399430

[ece374415-bib-0048] Tartanus, M. , W. Danelski , E. M. Furmańczyk , A. El Meziane , and E. Malusà . 2025. “Evaluation of Different Attractants for Monitoring and Mass Trapping of *Rhagoletis batava* (Hering) in Organic Sea Buckthorn Plantations.” Insects 16, no. 12: 1248. 10.3390/insects16121248. PMC12734082 41465686

[ece374415-bib-0049] Urbaneja‐Bernat, P. , J. González‐Cabrera , E. Hernández‐Suárez , and A. Tena . 2023. “Honeydew of HLB Vector, Trioza Erytreae, Increases Longevity, Egg Load and Parasitism of Its Main Parasitoid Tamarixia Dryi.” Biological Control 179: 105169. 10.1016/j.biocontrol.2023.105169.

[ece374415-bib-0050] van Achterberg, C. 2023. “Illustrated Key to the European Genera of Opiinae (Hymenoptera, Braconidae), With the Description of Two New Palaearctic Genera and Two New Species.” ZooKeys 1176: 79–115. 10.3897/zookeys.1176.104850. PMC10466209 37654982

[ece374415-bib-0051] Wei, S.‐J. , M. Shi , M. J. Sharkey , C. van Achterberg , and X.‐X. Chen . 2010. “Comparative Mitogenomics of Braconidae (Insecta: Hymenoptera) and the Phylogenetic Utility of Mitochondrial Genomes With Special Reference to Holometabolous Insects.” BMC Genomics 11: 371. 10.1186/1471-2164-11-371. PMC2890569 20537196

[ece374415-bib-0052] Wharton, R. A. 1997. “Generic Relationships of Opiine Braconidae (Hymenoptera) Parasitic on Fruit‐Infesting Tephritidae (Diptera).” Contributions of the American Entomological Institute 30, no. 3: 1–53.

[ece374415-bib-0053] Wu, Q. , C. van Achterberg , J.‐L. Tan , and X.‐X. Chen . 2016. “Review of the East Palaearctic and North Oriental *Psyttalia* Walker, With the Description of Three New Species (Hymenoptera, Braconidae, Opiinae).” ZooKeys 629: 103–151. 10.3897/zookeys.629.10167. PMC5126540 27920599

[ece374415-bib-0054] Wu, Z. , H. Chen , Y. Pan , et al. 2022. “Genome of *Hippophae rhamnoides* Provides Insights Into a Conserved Molecular Mechanism in Actinorhizal and Rhizobial Symbioses.” New Phytologist 235, no. 1: 276–291. 10.1111/nph.18017. 35118662

[ece374415-bib-0055] Xie, J. , Y. Chen , G. Cai , R. Cai , Z. Hu , and H. Wang . 2023. “Tree Visualization by One Table (tvBOT): A Web Application for Visualizing, Modifying and Annotating Phylogenetic Trees.” Nucleic Acids Research 51, no. W1: W587–W592. 10.1093/nar/gkad359. PMC10320113 37144476

[ece374415-bib-0056] Yang, D. , X. Hao , L. Jiang , et al. 2022. “The Complete Mitochondrial Genome of *Psyttalia incisi* (Silvestri, 1916) (Hymenoptera: Braconidae).” Mitochondrial DNA Part B: Resources 7, no. 6: 1038–1040. 10.1080/23802359.2022.2081942. PMC9225723 35756438

[ece374415-bib-0057] Yu, D. S. K. , C. van Achterberg , and K. Horstmann . 2016. “Taxapad 2016, Ichneumonoidea 2015.” Database on Flash‐Drive. Nepean, Ontario, Canada. http://www.taxapad.com.

[ece374415-bib-0058] Yu, W. , Y. Du , S. Li , et al. 2024. “Sea Buckthorn—Nutritional Composition, Bioactivity, Safety, and Applications: A Review.” Journal of Food Composition and Analysis 133: 106371. 10.1016/j.jfca.2024.106371.

[ece374415-bib-0059] Zeynalov, A. S. 2018. “Features of Bioecology of Sea Buckthorn Flies *Rhagoletis batava* Hering in the Central Non‐Chernozem Region of Russia.” Russian Agricultural Sciences 44, no. 4: 335–339. 10.3103/S1068367418040213.

[ece374415-bib-0060] Zhang, N. , J.‐M. Zhu , Y. Lu , A. Sattar , and G.‐S. Jin . 2017. “Population Dynamics and Biological Characteristics of *Rhagoletis batava* Hering in Burqin County.” Journal of Xinjiang Agricultural University 40, no. 6: 409–415.

[ece374415-bib-0061] Zhao, D. , T. Ye , F. Gao , et al. 2025. “PhyloSuite v2: The Development of an All‐In‐One, Efficient and Visualization‐Oriented Suite for Molecular Dating Analysis and Other Advanced Features.” iMeta 4: e70095. 10.1002/imt2.70095. PMC12747537 41472854

